# ICON: a randomized phase IIb study evaluating immunogenic chemotherapy combined with ipilimumab and nivolumab in patients with metastatic hormone receptor positive breast cancer

**DOI:** 10.1186/s12967-020-02421-w

**Published:** 2020-07-03

**Authors:** J. A. Kyte, N. K. Andresen, H. G. Russnes, S. Ø. Fretland, R. S. Falk, O. C. Lingjærde, B. Naume

**Affiliations:** 1 Department of Clinical Cancer Research, Oslo University Hospital, Oslo, Norway; 2 Department of Cancer Immunology, Oslo University Hospital, Oslo, Norway; 3 Department of Cancer Genetics, Oslo University Hospital, Oslo, Norway; 4 Department of Pathology, Oslo University Hospital, Oslo, Norway; 5 Oslo Centre for Biostatistics and Epidemiology, Oslo University Hospital, Oslo, Norway; 6 Department of Oncology, Oslo University Hospital, Oslo, Norway; 7 Institute of Clinical Medicine, University of Oslo, Oslo, Norway

**Keywords:** Breast cancer, Hormone receptor positive, Immunotherapy, Checkpoint inhibitor, Immunogenic cell death, PD-1, CTLA4, Anthracycline, Cyclophosphamide

## Abstract

**Background:**

Immunotherapy with checkpoint inhibitors (CPI) targeting PD-1 or CTLA-4 has emerged as an important treatment modality for several cancer forms. In hormone receptor positive breast cancer (HR + BC), this therapeutic approach is largely unexplored. We have started a clinical trial, ICON (CA209-9FN), evaluating CPI combined with selected chemotherapy in patients with metastatic HR + BC. The tumor lymphocyte infiltration is predictive for the effect of chemotherapy in BC. In ICON, we use anthracycline, which are considered as “immunogenic” chemotherapy, and low-dose cyclophosphamide, which has been reported to counter immunosuppressive cells.

**Methods:**

ICON is a randomized exploratory phase IIb study evaluating the safety and efficacy of combining nivolumab (nivo; anti-PD-1) and ipilimumab (ipi; anti-CTLA-4) with chemotherapy in subjects with metastatic HR + BC. Primary objectives are aassessment of toxicity and progression-free survival. The trial will enrol 75 evaluable subjects, randomized 2:3 into two arms (A:B). Patients in Arm A receive only chemotherapy, i.e. pegylated liposomal doxorubicin (PLD 20 mg/m^2^ intravenously every 2nd week) + cyclophosphamide (cyclo; 50 mg per day, first 2 weeks in each 4 week cycle). Patients in Arm B receive PLD + cyclo + ipilimumab (1 mg intravenously every 6th week) + nivolumab (240 mg intravenously every 2nd week). Patients in arm A will be offered ipi + nivo after disease progression.

**Discussion:**

ICON is among the first clinical trials combining chemotherapy with PD-1 and CTLA-4 blockade, and the first in BC. There is a strong preclinical rationale for exploring if anthracyclines, which are considered to induce immunogenic cell death, synergize with CPI, and for combining PD-1 and CTLA-4 blockade, as these checkpoints are important in different phases of the immune response. If the ICON trial suggests acceptable safety and provide a signal of clinical efficacy, further studies are warranted. The cross-over patients from Arm A receiving ipilimumab/nivolumab without concomitant chemotherapy represent the first BC cohort receiving this therapy. The ICON trial includes a series of translational sub-projects addressing clinically important knowledge gaps. These studies may uncover biomarkers or mechanisms of efficacy and resistance, thereby informing the development of novel combinatory regimes and of personalised biomarker-based therapy.

*Trial registration* NCT03409198, Jan 24th 2018; https://clinicaltrials.gov/ct2/show/record/NCT03409198

## Background

Immunotherapy with PD-1 and CTLA-4 inhibitors has shown remarkable clinical efficacy against several cancer forms [1–6] and now show activity in breast cancer [7–10]. This includes durable responses in metastatic breast cancer (mBC) patients, amid minimal adverse effects. Intriguingly, the host immune response is strongly predictive for the effect of chemotherapy (chemo) in BC [11]. We have started the trial ICON (CA209-9FN), a randomized phase IIb study evaluating Immunogenic chemotherapy COmbined with ipilimumab and Nivolumab in patients with hormone receptor positive metastatic BC (HR + mBC). Ipilimumab and nivolumab are monoclonal antibodies (mAbs) targeting CTLA-4 and PD-1, respectively. The strategy in the ICON trial is to release the brake on the chemo-induced immune response. We use pegylated liposomal doxorubicin (PLD) as the backbone of the chemotherapy, and combine with low-dose metronomic cyclophosphamide. These chemotherapeutic agents are considered to be potent inducers of immune responses. Further, the chosen drugs are accepted as 1st line therapy. This allows for including patients that have not received multiple lines of therapy and are may be more likely to respond.

PD-1 blockade has shown activity against metastatic breast cancer, but only in a minority of patients when used as monotherapy, and mainly in subjects with PD-L1 + triple negative BC (TNBC) [7]. There are limited data from HR + BC so far. Keynote 028 evaluated pembrolizumab monotherapy in heavily pretreated patients with HR + Her2 negative mBC [12]. The response rate was modest (12%), but some responses were durable (median 12 months). In the JAVELIN trial, also testing aPD1 as monotherapy in heavily pretreated mBC patients, only 2/110 subjects outside of the TNBC group recorded an objective response [13]. Tolaney and colleagues have conducted two phase II trials evaluating CPI combined with eribulin or radiotherapy against mHR + BC, where no efficacy of CPI was observed [14, 15]. The proportion of responders is greater when PD-1/PD-L1 blockers are given in the first line, rather than after several lines of chemotherapy (Schmid P ASCO 2017; Adams S ASCO 2017). The first randomized study comparing chemotherapy ± PD-L1 blockade against mBC, IMPASSION130, showed significant clinical benefit of adding atezolizumab (a-PD-L1) to taxanes, against triple negative breast cancer (TNBC) [8]. Based on this study, atezolizumab has been approved by the FDA and EMA in combination with taxanes for metastatic TNBC. Further, in early studies of preoperative therapy, the combination of PD-1 blockade and chemotherapy has produced a substantial increase in response rates, compared to chemotherapy alone, for both ER + /HER-2 negative and triple negative BC patients [9, 10]. Our study rationale is in line with these findings, combining checkpoint blockade with a carefully selected chemotherapy regimen, as 1st/2nd line metastatic treatment.

We utilize selected chemotherapy for inducing immunogenic cell death, which represents a personalized in vivo vaccination covering the entire repertoire of antigens expressed in each individual tumor. Such antigen release does not necessarily lead to an effective immune response. However, the chemotherapeutic agents chosen in ICON, doxorubicin and cyclophosphamide, induce “danger signals” that trigger the immune system [16]. In BC patients, these drugs are reported to induce a type I interferon response [11, 17], which suggests that they may turn an immunologically “cold” tumor into a tumor that responds to checkpoint blockade. There is also evidence from follow-up of BC patients, indicating that the survival effect of doxorubicin and cyclophosphamide depends on the host immune response [11, 18]. The IMPASSION130 trial used taxanes and did not show an effect of aPD-L1 in the PD-L1 negative group [8]. This highlights the need to explore if more immunogenic chemotherapy, as employed in ICON, can make “cold” tumors responsive to PD1-blockade. Data from recent trials in TNBC support the notion that anthracyclines may be superior to taxanes for this purpose, though no conclusion can yet be drawn. The TONIC trial addressed the issue of which chemotherapy to apply up front of PD1-blockade [19]. Here, induction therapy with doxorubicin yielded the highest response rate to nivolumab (anti-PD1), compared to other chemotherapy, radiation or no induction therapy. There was also biological evidence of immune activation in the tumor samples obtained after doxorubicin treatment. In the neoadjuvant setting, Keynote 522 showed significantly increased response rates for the group receiving aPD1 [10], when combined with chemotherapy, while the NEOTRIP trial did not show any benefit (Gianni et al. SABCS December 2019). The chemotherapy backbone in Keynote 522 contained anthracyclines and cyclophosphamide, while the NEOTRIP chemotherapy employed only taxanes.

ICON is to our knowledge the first clinical trial combining chemotherapy with PD-1 and CTLA-4 blockade in breast cancer. There is a strong preclinical rationale for combining PD-1 and CTLA-4 blockade, as these checkpoints are important in different phases of the immune response, and this drug combination has shown remarkable clinical efficacy in melanoma and lung cancer [2, 6]. In breast cancer, animal studies have demonstrated a substantial advantage by adding anti-CTLA-4 to anti-PD-1 and chemotherapy [20]. Taken together, there is a strong rationale for synergy between doxorubicin/cyclophosphamide and PD-1/CTLA-4 blockade [21].

## Methods

### Study design

**Fig. 1:**
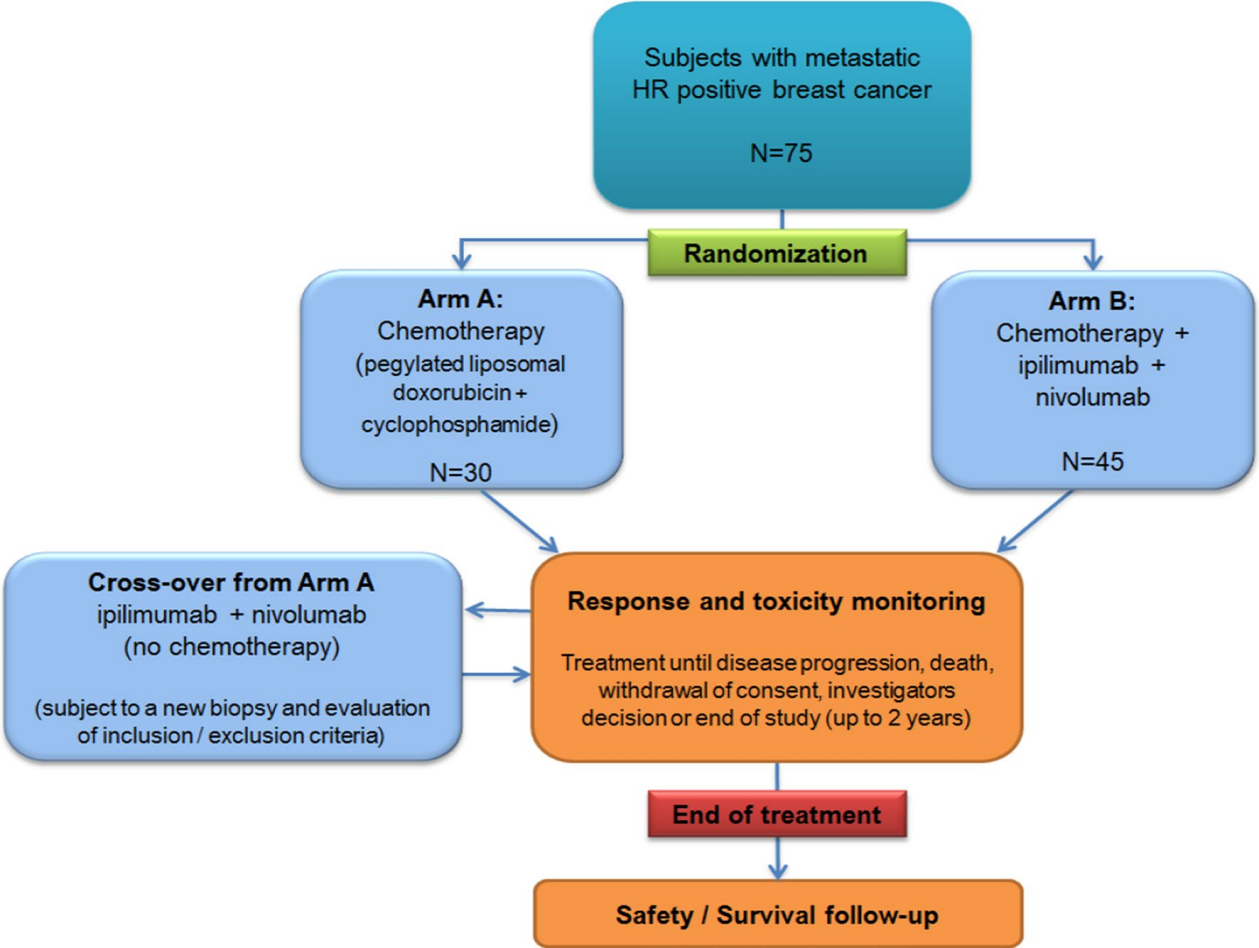
Study design

This is a randomized phase IIb study evaluating the safety and efficacy of combining nivolumab and ipilimumab with immunogenic chemotherapy in subjects with metastatic HR + breast cancer (Fig. 1). The patients will be randomized 2:3 into two arms (A:B):

- Arm A: Chemotherapy only (pegylated liposomal doxorubicin + cyclophosphamide)
- Arm B: Chemotherapy + ipilimumab + nivolumab

- Upon progression, patients may continue study treatment until loss of clinical benefit.
- The patients in arm A will be offered nivo/ipi (without chemotherapy) after disease progression.

### Objectives

**Table:** 

Primary
Assessment of toxicity
Assessment of progression-free survival (PFS)
Secondary
Assessment of clinical response in ipi/nivo/chemo group compared to chemo only group: Objective tumor response rate (ORR), duration of response (DR), durable tumor response rate (DRR; > 6 months), clinical benefit rate (CBR), overall survival (OS)
Assessment of toxicity of ipi/nivo (without chemotherapy) in cross-over arm
Assessment of ORR, DR, DRR, CBR, PFS and OS in cross-over arm receiving ipi/nivo (without chemotherapy)
Assessment of PD-L1 expression, mutation load and immune gene expression as biomarkers for clinical response
Characterization of changes in immunological milieu induced by the combination therapy (ipi/nivo/chemo), as compared to chemo only, and by ipi/nivo without concomitant chemotherapy
Comparison of clinical and biological response in molecular subtypes of breast cancer
Assessment of patient reported outcomes, as measured by the Chalder Fatigue Questionnaire (FQ), an 11 point Numerical Rating Scale (NRS) for pain intensity and EORTC QLQ-C15-PAL
Assessment of immunological response
Identification of biomarkers for clinical response, toxicity and immune response
Characterization of tumor evolution induced by the combination therapy (ipi/nivo/chemo), as compared to chemo only, and by ipi/nivo without concomitant chemotherapy

### Study treatment

- Nivolumab 240 mg intravenously (i.v.) every 2nd week until disease progression or for a maximum of 24 months (flat dose of 240 mg recommended by BMS)
- Ipilimumab 1 mg i.v. every 6th week until disease progression (maximum 24 months)
  - If toxicity unacceptable, apply dose-1 level for ipilimumab of 1 mg every 12th week

- Pegylated liposomal doxorubicin (PLD; Caelyx) 20 mg/m^2^ i.v. every 2nd week.
- Cyclophosphamide tablets 50 mg per day, daily for 2 first weeks in each 4 week cycle.

#### Rationale for immunotherapy regime

The combination of CTLA-4 and PD-1 checkpoint blockade has shown high efficacy in melanoma, but also a relatively high frequency of grade 3 and 4 adverse events. This toxicity mainly correlates with the Ipilimumab dosage. We therefore use a low dosage of Ipilimumab (1 mg/kg vs 3 mg/kg in melanoma) and prolonged intervals between treatments (6 weeks). This regime has in recent studies shown good efficacy and a much safer toxicity profile [6, 22].

#### Rationale for chemotherapy regime

Doxorubicin and cyclophosphamide are considered as powerful inducers of immunogenic cell death and thus attractive for combination with immune checkpoint inhibitors. There is also some evidence from breast cancer patients, supporting this notion, as outlined above. In ICON, we use a pegylated liposomal formulation of doxorubicin to minimize the adverse effects of anthracyclines on the heart and allow for continued treatment beyond otherwise mandatory anthracycline limits. The possibility of long term treatment is important in order to appropriately test checkpoint inhibitors, as these drugs are known to induce durable responses in other patient groups. Pegylated liposomal doxorubicin is, moreover, administered without any need for corticosteroids, which is desirable for immunotherapy.

We employ a semi-metronomic PLD regime, rather than a high dose regime administered every 3rd/4th week. This dosing regime allows for improved possibility to control toxicity, and to limit profound leukopenia, which may increase the ability of the effector immune cells to respond.The standard pegylated liposomal doxorubicin dose for breast cancer is 40–50 mg/m^2^ every 4th week. In Norway, the most widely used dose is 40 mg/m^2^. The dose chosen in this study is expected to be well tolerated, as the 40 mg/m^2^ is divided into two doses of 20 mg/m^2^ given every 2nd week. We regard the chemotherapy regime as appropriate therapy for this patient group, without nivo/ipi. The regime is applicable to most metastatic patients with ECOG 0–1, while also being sufficiently potent to suit those with an excellent performance status. Anthracyclines are routinely administered at intervals ranging from one to four weeks in metastatic breast cancer patients. There is no firm basis for recommending epirubicin/cyclophosphamide (EC60) every 3rd week, PLD (usually every 4th week) or epirubicin weekly, except for an individualized consideration of toxicity and the consideration to limit travelling/time in hospital. Some studies in breast cancer have used pegylated liposomal doxorubicin at 15–30 mg/m^2^ every 2nd week, or in combination with cyclophosphamide (500 mg/m^2^), and 5-fluorouracil (500 mg/m^2^) every 3rd week [23–26]. A dose of 20 mg/m^2^ has been well tolerated in combination with cyclophosphamide (50 mg/day) even in fragile, older patients [27] and is also tolerated by HIV positive patients with Kaposi sarcoma.

Tregs and MDSCs represent important mediators of tumor tolerance and may oppose the effect of nivo/ipi. The metronomic cyclophosphamide dosage chosen in the present study has been widely used to counter Tregs and MDSCs [28], and has also been applied against metastatic breast cancer [25]. It is considered safe, as it has been combined with multiple other chemotherapeutic agents without causing important toxicity [25]. We include 14-day intervals without cyclophosphamide to allow for unsuppressed T cell proliferation and activity, which may be important for the nivo/ipi effect.

Previous endocrine and targeted therapy is not mandatory, but only patients considered to need chemotherapy should be considered for the trial. In most cases, this means that the patients have progressed on endocrine therapy and cycline dependent kinase inhibitors before entering the trial.

### Selected inclusion criteria

1. Metastatic hormone receptor positive breast cancer (primary or recurrent), defined as ER +  > 1% in metastatic biopsy (archival material or study biopsy) or cytology and HER2 negative in the last biopsy or cytology evaluable for HER2.
2. Adequate core or excisional study biopsy of a tumor lesion. At least one FFPE biopsy must me obtained at screening, and considered by macroscopic examination to represent tumor, but no verification by pathologist is required. Cytology is not sufficient.
3. Measurable metastatic disease according to RECIST.
4. Eastern Cooperative Oncology Group (ECOG) performance status of 0 or 1.
5. Signed Informed Consent Form.
6. Women or men aged ≥ 18 years.
7. A minimum of 12 months from adjuvant/neoadjuvant chemotherapy with anthracyclines to relapse of disease.
8. A maximum of one previous line with chemotherapy in the metastatic setting.
9. Previous endocrine and targeted therapy is allowed, including cycline dependent kinase inhibitors.
10. Adequate organ function as defined in the protocol.

### Selected exclusion criteria

1. Malignancies other than breast cancer within 5 years prior to randomization, with the exception of those with a negligible risk of metastasis or death and treated with expected curative outcome.
2. Spinal cord compression not definitively treated with surgery and/or radiation, or previously diagnosed and treated spinal cord compression without evidence that disease has been clinically stable for > 2 weeks prior to randomization.
3. Known CNS disease, except for asymptomatic CNS metastases, provided all of the following criteria are met:
  - Measurable disease outside the CNS.
  - Asymptomatic for CNS disease > 4 weeks.
  - No ongoing requirement for corticosteroids as therapy for CNS disease.
  - No radiation of brain lesions within 2 weeks prior to randomization.
  - No leptomeningeal disease.
4. Uncontrolled pleural effusion, pericardial effusion, or ascites.
5. Pregnant or breastfeeding.
6. Received treatment with immune checkpoint modulators.
7. Received treatment with systemic corticosteroids or other systemic immunosuppressive medications within 2 weeks prior to randomization, or anticipated requirement for systemic immunosuppressive medications during the trial.
  - Patients who have received acute, low-dose, systemic immunosuppressant medications (e.g., a one-time dose of dexamethasone for nausea) may be enrolled in the study.
  - Patients with a history of allergic reaction to IV contrast requiring steroid pre-treatment should have baseline and subsequent tumor assessments performed using MRI.
  - The use of inhaled corticosteroids for chronic obstructive pulmonary disease, mineralocorticoids (e.g., fludrocortisone) for patients with orthostatic hypotension, and low-dose supplemental corticosteroids for adrenocortical insufficiency are allowed.
8. Received anti-cancer therapy (medical agents or radiation) within 2 weeks prior to study Cycle 1, Day 1. Palliative radiotherapy for bone lesions is allowed up to 7 days before start of therapy.

### Outcome measures

#### Safety outcome measures

The safety outcome measures will be evaluated in the ITT population, as follows:

- Incidence, nature, and severity of adverse events graded according to NCI CTCAE v4.0.
- Changes in vital signs, physical findings, and clinical laboratory results.

#### Efficacy outcome measures

The PFS is defined as the time from randomization to the time of radiographic progression (as assessed by RECIST v1.1) or death from any cause during the study. Data for patients with a PFS event who missed two or more assessments scheduled immediately prior to the date of the PFS event will be censored at the last tumor assessment prior to the missed visits. If no tumor assessment was performed after randomization, data will be censored at the date of randomization + 1 day. Clinical deterioration without objective radiological evidence will not be considered as documented disease progression. The primary efficacy outcome measure (PFS) is to be assessed in patients evaluable per protocol (PP).

The secondary efficacy outcome measures will be assessed in the PP population, ITT population and in the PD-L1-positive subpopulation. The ITT population is defined as a full analysis set (FAS). The FAS is defined as all patients that have started therapy with at least one of the IMPs, and where data on the relevant endpoint is obtained. The safety will be evaluated in the ITT (FAS) population.

### Safety and management of adverse events

Safety will be evaluated in this study through the monitoring of all serious and non-serious adverse events defined and graded according to NCI CTCAE v4.0. Patients will be assessed for safety (including laboratory values). Laboratory values must be reviewed prior to each administration of IMP.

General safety assessments will include serial interval histories, physical examinations, and specific laboratory studies, including serum chemistries and blood counts.

During the study, patients will be closely monitored for the development of any adverse events, including signs or symptoms of autoimmune conditions and infection. All serious adverse events and protocol-defined events of special interest will be reported in an expedited fashion.

Patients will be followed for AE for 30 days, and for SAE and AESI that are believed to be related to the study drug for 100 days, following their last dose of study drug. Patients who have an ongoing study drug related adverse event upon study completion or at discontinuation from the study will be followed until the event has resolved to baseline grade, the event is assessed by the investigator as stable, the patient is lost to follow-up, the patient withdraws consent, or until it has been determined that study treatment or participation is not the cause of the adverse event.

For recommendations on the management of adverse events related to nivolumab or ipilimumab, the protocol refers to the last updated versions of the Investigator´s Brochures (IB) for nivolumab and ipilimumab. If, in the judgment of the investigator, the patient is likely to derive clinical benefit from receiving study therapy that is not in accordance with the recommendations in the IB, this must be approved by the Sponsor before such therapy is given, and the patient must be appropriately informed.

### Dose modification

#### General notes regarding dose modification

- If, in the opinion of the investigator, a toxicity is considered to be due to one/two/three component(s) of the treatment (i.e. nivo, ipi, cyclophosphamide or PLD) and the dose of that/those component(s) is/are delayed or modified in accordance with the guidelines below, the other component(s) may be administered if there is no contraindication.
- When treatment is temporarily interrupted because of toxicity, the treatment cycles will be restarted such that the nivolumab and chemotherapy infusions remain synchronized.
- If it is anticipated that chemotherapy will be delayed by ≥ 10 days, then nivo/ipi should be given without the chemotherapy if there is no contraindication.
- The treating physician may use discretion in modifying or accelerating the dose modification guidelines described below depending on the severity of toxicity and an assessment of the risk versus benefit for the patient, with the goal of maximizing patient compliance and access to supportive care.

#### Nivolumab/ipilimumab dose modification

There will be no dose reduction for nivolumab in this study. If a patient experiences a grade 3 or 4 adverse event considered probably related to ipilimumab, the ipilimumab dosing interval should be extended to every 12 week. The ipilimumab dose should be kept at 1 mg/kg. Patients may temporarily suspend study treatment with Nivolumab/Ipilimumab if they experience an adverse event that requires a dose to be held.

- If nivolumab is held because of adverse events for > 42 days (6 weeks) beyond the last dose, then the patient will be discontinued from nivolumab treatment. If, in the judgment of the investigator, the patient is likely to derive clinical benefit from resuming nivolumab after a hold > 42 days, study drug may be restarted with the approval of the Sponsor. If a patient must be tapered off steroids used to treat adverse events, nivolumab may be held for > 42 days until steroids are discontinued or reduced to prednisone dose (or dose equivalent) ≤ 10 mg/day. The acceptable length of interruption will depend on agreement between the investigator and the Sponsor.
- If ipilimumab is held because of adverse events for > 126 days (18 weeks) beyond the last dose, then the patient will be discontinued from ipilimumab treatment. If, in the judgment of the investigator, the patient is likely to derive clinical benefit from resuming ipilimumab after a hold > 126 days, study drug may be restarted with the approval of the Sponsor. If a patient must be tapered off steroids used to treat adverse events, ipilimumab may be held for > 126 days until steroids are discontinued or reduced to prednisone dose (or dose equivalent) ≤ 10 mg/day. The acceptable length of interruption will depend on agreement between the investigator and the Sponsor.
- Dose interruptions for reason(s) other than adverse events, such as surgical procedures, may be allowed with Sponsor approval. The acceptable length of interruption will depend on agreement between the investigator and the Sponsor.
- Patients who discontinue treatment with either nivolumab or ipilimumab due to AE may continue treatment with the other IMPs.

#### Chemotherapy dose modification

Pegylated liposomal doxorubicin will be administered in accordance with standard procedures and established practice at the study hospitals, including criteria for hematological counts. Cardiac function will be monitored if clinically indicated and according to routine practice. Dose reduction and delay of treatment is allowed when performed in accordance with standard practice and in line with the guidelines given in the pegylated liposomal doxorubicin Product Information, as listed at ema.europa.eu. This includes guidelines on the management of stomatitis, palmar–plantar erythrodysesthesia and haematological toxicity.

The following adjustments to the standard guidelines apply: A grade 3 or 4 lymphopenia is to be handled as the corresponding grade of neutropenia. The administration of pegylated liposomal doxorubicin is withheld until the lymphopenia and neutropenia resolves to grade ≤ 2, i.e. lymphocyte count ≥ 0.5 × 109/L and neutrophil count ≥ 1.0 × 109/L. For grade 2 neutropenia or lymphopenia, the dose of pegylated liposomal doxorubicin should be reduced to 15 mg/m^2^.

Pegylated liposomal doxorubicin may be reduced in dose by up to 25%, i.e. to 15 mg/m^2^ or paused with up to two weeks when considered necessary by the investigator. If the investigator considers that a more extended pause is necessary, the investigator should consult a member of the Study Leadership. Dose reductions below 15 mg/m^2^ are not allowed.

Metronomic cyclophosphamide, as used in the study, is expected to be well tolerated. If considered necessary by the investigator, the drug may be omitted for up to two weeks. If the investigator considers that a more extended pause is necessary, the investigator should consult a member of the Study Leadership.

### Sample collection/biobanking

Samples are collected before, during and after therapy (time of progression/treatment discontinuation). Some of the biobanking procedures are not performed at all study centers. The following samples are collected:

- Tumor biopsies collected at screening, after 8 weeks, 6 months and at time of treatment discontinuation. Only the screening biopsy is mandatory. If sufficient tissue is available, three biopsies will be obtained at each time point, and prioritized in the following order:
  - FFPE tissue.
  - Snap-frozen tumor biopsies.
  - Fresh tumor cells/ tumor infiltrating lymphocytes freshly prepared into cell suspension.

- Blood samples collected pre-, during and post-therapy:

- Peripheral blood mononuclear cells, processed with gradient centrifugation and frozen on liquid nitrogen (up to 8 time points).
- Plasma and serum, separated and frozen (up to 13 time points).
- Circulating tumor cells at screening, 4 weeks and at time of treatment discontinuation.

- Urine samples collected at screening, 8 weeks and at time of treatment discontinuation.
- Faecal samples collected at screening and after 8 weeks of therapy.

### Statistics

A descriptive analysis of demographics, medical history, and clinical data will be performed.

The ITT population is defined as a full analysis set (FAS). The FAS is defined as all patients that have started therapy with at least one of the IMPs, and where data on the relevant endpoint is obtained. The safety will be evaluated in the ITT (FAS) population.

The primary efficacy analyses will be performed on the PP population. Secondary efficacy analyses will be performed on the PD-L1 positive subpopulation and on the ITT (FAS) population.

The primary efficacy analysis will be an analysis of progression free survival (PFS) in the combination arm, compared to the control group. PFS is, defined as the time from randomization to the occurrence of disease progression or death from any cause, whichever occurs first. Data for patients without disease progression or death will be censored at the last tumor assessment date. Data for patients with a PFS event who missed two or more assessments scheduled immediately prior to the date of the PFS event will be censored at the last tumor assessment prior to the missed visits. If no tumor assessment was performed after randomization, data will be censored at the date of randomization + 1 day. Clinical deterioration without objective radiological evidence will not be considered as documented disease progression. Comparison between treatment arms will also be given by HR for disease progression or death using a Cox proportional hazards model. The HR will be adjusted for the factors listed below. The 95% CI for the HR will be provided. Kaplan–Meier methodology will be used, and Kaplan–Meier curves will be produced.

Overall survival (OS) will be calculated from time of randomization until death. Patients alive at the time of data analysis will be treated as censored. OS will be estimated by the Kaplan Meier method.

Exploratory analyses will be carried out to evaluate the data of the immunological and molecular analyses (e.g. biomarker studies) carried out. The statistical analyses will be dependent on the biological factors investigated and the analysis methodology used, and will be defined separately for each molecular study.

We expect to reach the data-driven time point for PFS-analysis (70 PFS events in the PP population) approximately 3 years after the study opens. If this is not met within 24 months after inclusion of the last patient, the PFS-analysis will be performed at this time point.

The primary data analysis will be performed on the PP population and analyzed according to the following factors:

- Tumor PD-L1 status
- Disease free interval between end of (neo)adjuvant chemotherapy or surgery, whichever was last, and relapse
- Time from diagnosis of metastatic disease to start of therapy in the ICON-study Prior chemotherapy against metastatic disease (no previous chemo vs. previous chemo). Chemotherapy given in the neoadjuvant/adjuvant setting is not to be considered in this analysis
- Sites of metastases
- Molecular breast cancer profile, including PAM50 subtype, and immune gene profile

Exploratory analyses will be carried out to evaluate data from translational studies. Here, statistical methods will be defined separately for each study, as advised by the statisticians.

#### Statistical considerations regarding sample size and randomization ratio

The phase II study cannot be powered to demonstrate a statistically significant (p < 0.05) clinical effect. If the study suggests acceptable toxicity and potential clinical benefit, a larger randomized study will be warranted. We plan to conduct a phase II study with 75 patients (45 patients in the nivo-chemo arm, 30 patients in the chemo-only arm).

The number of 75 patients and the randomization ratio of 3:2 were based on the following considerations given below.

**Table:** Expected PFS for the control group, receiving only chemotherapy

Months	Progression-free proportion
5	50%
12	25%
20	5%

#### Statistical power calculations for the primary endpoint PFS

A two-sided hypothesis test was performed with a 10% significance level and a desired power of 80%. The test was NOT performed to define a number of patients where a significant clinical effect (p < 0.05) could be determined, as this is not the aim of a phase II trial. Rather, the calculation was done to illustrate what we can expect to observe within a realistic number of patients for a phase II trial and to inform the choice of randomisation ratio. For this purpose, we choice a significance level of 10% and performed the test for a sample size of 60, 75 and 80 patients. The calculation for 75 patients is given below (Table 1).

**Table 1:** Statistical power with 75 patients

Randomisation	Surv1	Surv2	HR
1:1	0.05	0.208	0.52
2:3	0.05	0.198	0.54
1:2	0.05	0.198	0.54

Surv1 is the survival probability in the control group at the end of the study. Surv2 is the survival probability in the experimental group. *HR*, hazard ratio, effect size of the experimental to the control group

The power calculation indicates that a randomization ratio of 2:3 or 1:2 is preferable to 1:1. A ratio of 2:3 is chosen rather than 1:2 in order to increase the statistical power for collateral research analyses.

The biomarker research program aims at identifying which patients benefit from treatment and may inform the design of a subsequent randomized trial. The suggested number of patients will allow for meaningful statistical comparisons of biological/immunological data, and comparison with data from our previous studies and the OUS breast cancer biobank.

## Discussion

### Study organization and timeline

Oslo University Hospital, Oslo, Norway, is the study sponsor. We have established three study centers in Norway (Oslo University Hospital, Stavanger University Hospital, SSHF Kristiansand) and three in Belgium (Institute Jules Bordet Brussels, Cliniques Saint Luc Brussels, CHU UCL Namur). The study opened February 2018, expanded to more sites in 2019 and has included 63 patients as of March 1st 2020. We estimate a need to recruit 80 patients, to obtain the required 75 evaluable patients per protocol.

### Comments on study design

The clinical development of novel drugs as add-ons to established therapy is challenging, as conventional one-armed phase I/II studies may not be suitable for providing information on the effect and toxicity of the add-on drug. In many patient populations, like the HR + BC investigated in the ICON-trial, historic controls are heterogeneous and of limited value. In our case, neither the effect nor the toxicity of adding checkpoint inhibitors to chemotherapy could be properly assessed in a one-armed study. On the other hand, a full-scale phase III trial, powered to show clinical efficacy with a *p* < 0.05, is not warranted, too resource demanding and ethically problematic, until basic clinical data have been generated. In the ICON-study, we therefore chose a randomized phase IIb design, with a limited number of patients. The aim is to assess the toxicity of the CPIs as add-ons to the chemotherapy, and provide leads on potential clinical efficacy in the overall HR + BC patient population, as well as in subgroups identified by biomarkers. Accordingly, the study was not powered to demonstrate a statistically significant clinical effect. If the study suggests acceptable toxicity and potential clinical benefit, a larger randomized study will be warranted.

The biomarker analyses and other translational projects included in ICON may inform the selection of patient subgroups for later studies and allow for more personalised therapy. Moreover, the ICON trial is designed to address the question of which chemotherapy should be added to checkpoint inhibitors. It is important to point out that the hypothesised beneficial effects of anthracyclines (immunogenic cell death) and low-dose cyclophosphamide (counter Tregs/MDSCs) on the immune milieu have not yet been convincingly re-produced in patient cohorts. A critical evaluation of these hypotheses is among the listed objectives in the ICON trial, and may have implications for the choice of chemotherapy for combination with checkpoint inhibitors in future studies, both in BC and other cancer forms.

## Data Availability

Not applicable.

## Abbreviations

BC: Beast cancer
CBR: Clinical benefit rate
CPI: Checkpoint inhibitor
CNS: Central nervous system
CR: Complete response
CTC: Circulating tumor cells
CTCAE: Common terminology criteria for adverse event
CTLA-4: Cytotoxic T lymphocyte antigen 4
DOR: Duration of objective response
DR: Duration of response
DRR: Durable tumor response rate
ECOG: Eastern cooperative oncology group
EMA: European medicines agency
ER: Estrogen receptor
FAS: Full analysis set
HER2: Human epidermal growth factor receptor 2
HR: Hazard ratio
ICH: International conference on harmonization
IHC: Immunohistochemistry
IMP: Investigational medicinal product
ISH: In situ hybridization
ITT: Intention to treat
IV: Intravenous
MDSC: Myeloid-derived suppressor cells
ORR: Overall response rate
OS: Overall survival
PBMC: Peripheral blood mononuclear cell
PD: Progressive disease
PD-1: Programmed death 1
PD-L1: Programmed death ligand-1
PFS: Progression-free survival
PLD: Pegylated liposomal doxorubicin
PP: Per protocol
PR: Progesterone receptor
QLQ: Quality of life questionnaire
SD: Stable disease

## References

[1] Robert C (2014). Anti-programmed-death-receptor-1 treatment with pembrolizumab in ipilimumab-refractory advanced melanoma: a randomised dose-comparison cohort of a phase 1 trial. Lancet.

[2] Larkin J (2015). Combined nivolumab and ipilimumab or monotherapy in untreated melanoma. N Engl J Med.

[3] Gibney GT (2015). Safety, correlative markers, and clinical results of adjuvant nivolumab in combination with vaccine in resected high-risk metastatic melanoma. Clin Cancer Res.

[4] Rizvi NA (2015). Cancer immunology. Mutational landscape determines sensitivity to PD-1 blockade in non-small cell lung cancer. Science.

[5] Ferris RL (2016). Nivolumab for recurrent squamous-cell carcinoma of the head and neck. N Engl J Med.

[6] Hellmann MD (2018). Nivolumab plus ipilimumab in lung cancer with a high tumor mutational burden. N Engl J Med.

[7] Emens LA (2018). Breast cancer immunotherapy: facts and hopes. Clin Cancer Res.

[8] Schmid P (2018). Atezolizumab and nab-paclitaxel in advanced triple-negative breast cancer. N Engl J Med.

[9] Nanda R (2020). Effect of pembrolizumab plus neoadjuvant chemotherapy on pathologic complete response in women with early-stage breast cancer: an analysis of the ongoing phase 2 adaptively randomized I-SPY2 trial. JAMA Oncol.

[10] Schmid P (2020). Pembrolizumab for early triple-negative breast cancer. N Engl J Med.

[11] Sistigu A (2014). Cancer cell-autonomous contribution of type I interferon signaling to the efficacy of chemotherapy. Nat Med.

[12] Rugo HS (2018). Safety and antitumor activity of pembrolizumab in patients with estrogen receptor-positive/human epidermal growth factor receptor 2-negative advanced breast cancer. Clin Cancer Res.

[13] Dirix LY (2018). Avelumab, an anti-PD-L1 antibody, in patients with locally advanced or metastatic breast cancer: a phase 1b JAVELIN Solid Tumor study. Breast Cancer Res Treat.

[14] Barroso-Sousa R (2020). A phase II study of pembrolizumab in combination with palliative radiotherapy for hormone receptor-positive metastatic breast cancer. Clin Breast Cancer.

[15] Tolaney SM (2019). Randomized phase II study of eribulin mesylate (E) with or without pembrolizumab (P) for hormone receptor-positive (HR+) metastatic breast cancer (MBC). J Clin Oncol.

[16] Bezu L (2015). Combinatorial strategies for the induction of immunogenic cell death. Front Immunol.

[17] Kroemer G (2015). Natural and therapy-induced immunosurveillance in breast cancer. Nat Med.

[18] Apetoh L (2007). Toll-like receptor 4-dependent contribution of the immune system to anticancer chemotherapy and radiotherapy. Nat Med.

[19] Voorwerk L (2019). Immune induction strategies in metastatic triple-negative breast cancer to enhance the sensitivity to PD-1 blockade: the TONIC trial. Nat Med.

[20] Nolan E (2017). Combined immune checkpoint blockade as a therapeutic strategy for BRCA1-mutated breast cancer. Sci Transl Med.

[21] Pfirschke C (2016). Immunogenic chemotherapy sensitizes tumors to checkpoint blockade therapy. Immunity.

[22] Goldman JW (2017). Nivolumab (N) plus ipilimumab (I) as first-line (1L) treatment for advanced (adv) NSCLC: 2-yr OS and long-term outcomes from CheckMate 012. J Clin Oncol.

[23] Rau KM (2015). Pegylated liposomal doxorubicin (Lipo-Dox(R)) combined with cyclophosphamide and 5-fluorouracil is effective and safe as salvage chemotherapy in taxane-treated metastatic breast cancer: an open-label, multi-center, non-comparative phase II study. BMC Cancer.

[24] Jehn CF (2016). Biweekly pegylated liposomal doxorubicin (Caelyx) in heavily pretreated metastatic breast cancer: a phase 2 study. Clin Breast Cancer.

[25] Munzone E, Colleoni M (2015). Clinical overview of metronomic chemotherapy in breast cancer. Nat Rev Clin Oncol.

[26] Rossi D (2008). Neoadjuvant chemotherapy with low dose of pegylated liposomal doxorubicin plus weekly paclitaxel in operable and locally advanced breast cancer. Anticancer Drugs.

[27] Dellapasqua S (2011). Pegylated liposomal doxorubicin in combination with low-dose metronomic cyclophosphamide as preoperative treatment for patients with locally advanced breast cancer. Breast.

[28] Ghiringhelli F (2007). Metronomic cyclophosphamide regimen selectively depletes CD4+CD25+ regulatory T cells and restores T and NK effector functions in end stage cancer patients. Cancer Immunol Immunother.

